# Ventilation Series Similarity: A Study for Ventilation Calculation Using Deformable Image Registration and 4DCT to Avoid Motion Artifacts

**DOI:** 10.1155/2017/9730380

**Published:** 2017-09-17

**Authors:** Geoffrey G. Zhang, Kujtim Latifi, Vladimir Feygelman, Kuei-Ting Chou, Tzung-Chi Huang, Thomas J. Dilling, Bradford A. Perez, Eduardo G. Moros

**Affiliations:** ^1^Radiation Oncology, Moffitt Cancer Center, Tampa, FL, USA; ^2^Department of Biomedical Imaging and Radiological Science, China Medical University, Taichung City, Taiwan; ^3^Department of Bioinformatics and Medical Engineering, Asia University, Taichung City, Taiwan

## Abstract

The major problem with ventilation distribution calculations using DIR and 4DCT is the motion artifacts in 4DCT. Quite often not all phases would exhibit mushroom motion artifacts. If the ventilation series similarity is sufficiently robust, the ventilation distribution can be calculated using only the artifact-free phases. This study investigated the ventilation similarity among the data derived from different respiration phases. Fifteen lung cancer cases were analyzed. In each case, DIR was performed between the end-expiration phase and all other phases. Ventilation distributions were then calculated using the deformation matrices. The similarity was compared between the series ventilation distributions. The correlation between the majority phases was reasonably good, with average SCC values between 0.28 and 0.70 for the original data and 0.30 and 0.75 after smoothing. The better correlation between the neighboring phases, with average SCC values between 0.55 and 0.70 for the original data, revealed the nonlinear property of the dynamic ventilation. DSC analysis showed the same trend. To reduce the errors if motion artifacts are present, the phases without serious mushroom artifacts may be used. To minimize the effect of the nonlinearity in dynamic ventilation, the calculation phase should be chosen as close to the end-inspiration as possible.

## 1. Introduction

Lung function is often characterized by perfusion and ventilation. Clinically, the major imaging modalities for ventilation are SPECT [1] and PET [2]. Recently, deriving lung ventilation distribution from 4-dimensional Computed Tomography (4DCT) using deformable image registration (DIR) has been developed [3–5]. One of the advantages of this approach is its higher spatial resolution compared to nuclear medicine scans [6]. It has been shown that this technique agrees reasonably well with other methods, such as SPECT, Xenon-enhanced dynamic CT, and PET [7–10]. Although ventilation distribution by Xenon-enhanced dynamic CT can achieve similar resolution, because of the dynamic scanning, only a part of the lung can be covered in a ventilation scan, and the technique itself is more complicated compared to 4DCT [7, 10]. Since 4DCT is already widely used in thoracic cancer radiotherapy treatment planning, in principle, using the 4DCT/DIR technique, one could determine regions of high lung ventilation in thoracic cancer patients and attempt to spare them in radiotherapy treatment planning, without the need for an additional imaging procedure [11]. Therefore, 4DCT-based ventilation assessment represents a relatively simple and low cost way to further personalized radiation therapy treatment planning. The ventilation calculation method using 4DCT is being clinically investigated in lung disease detection [12], radiotherapy treatment planning studies [11, 13], and assessment of radiotherapy response [14].

However, the 4DCT-DIR technique is susceptible to noise and artifacts in the 4DCT data [15, 16]. The most common artifacts in a 4DCT are caused by organ motion. One of the frequently seen artifacts is called a mushroom artifact because of its shape [17]. The mushroom artifacts are caused by the diaphragm truncation and duplication in some phases of a 4DCT, due to the irregular diaphragm motion during the scan. When a 4DCT dataset is used in DIR, irregularities in the deformation matrix would occur in the artifact region, which would in turn cause errors in the derived ventilation distribution.

**Figure 1:**
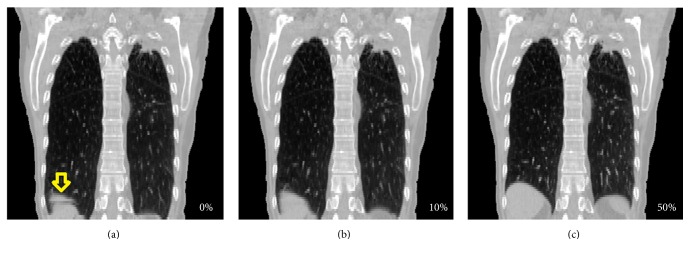
Mushroom artifacts can be seen at the diaphragm region in the right lung on 0% phase (panel (a), indicated by an arrow) but not on others (panels (b) and (c)).

A set of 4DCT data consists of a number of respiratory phases, typically 10. By convention, phase 0% is the end-inspiration phase while phase 50% is the end-expiration one. Ventilation distribution is usually derived from the deformation matrix between phases 0% and 50%, which represent the largest respiration difference. Even when mushroom artifacts (Figure 1) are seen, they are not necessarily present on every phase. One way to alleviate the mushroom artifact problem is to train the patient to breath regularly and repeat the scan. Another option, when rescanning is not possible, is to use only the phases without mushroom artifacts to perform the DIR and subsequent ventilation calculations. Obviously this approach is only feasible if derived ventilation distribution data are sufficiently robust with respect to the choice of respiratory phases used for calculations. Thus the goal of the current paper is to investigate stability of the derived ventilation data when different phases are used in the ventilation calculation.

Part of the results of this study was presented at The International Congress on Clinical Trials in Oncoloty and Hemato-Oncology, March 16-17, 2017, London, UK.

## 2. Materials and Methods

### 2.1. Patient Data

Fifteen lung cancer patients' 4DCT datasets were used in this study following a protocol approved by the institutional review board. No dataset used in the study had pronounced mushroom artifacts, to streamline the ventilation similarity comparison between the different phases. No other inclusion/exclusion criteria were applied. The case shown in Figure 1 was also analyzed as an example of a mushroom artifact being present, to demonstrate the practicality of the method. Each 4DCT dataset consisted of 10 respiratory phases, with axial image pixel size of about 1 × 1 mm^2^ and 3 mm slice thickness.

### 2.2. Deformable Image Registration

Based on a previous DIR selection study [18], the Diffeomorphic Morphons (DM) method [19] was used to register the end-expiration phase to all others in each 4DCT dataset.

### 2.3. Ventilation

The geometric, or Δ*V*, method was used. It directly calculates the local volume change using the deformation matrix resulting from the DIR [3]. In the deformation matrix, a 3-dimensional (3D) displacement vector is recorded for each voxel between the two registered 3D image sets. The local volume change is calculated based on the neighboring voxel displacements. Ventilation for each voxel is then calculated as

(1) $$\begin{array}{c} P=\frac{\Delta V}{V_{ex}}, \end{array}$$

where Δ*V* is the volume change between the involved respiration phases and *V*_ex_ is the initial volume at expiration [20].

Each ventilation distribution calculation involves two respiration phases. The full ventilation is calculated using the end-inspiration (0%) and the end-expiration (50%) phases, which is denoted here as *V*_50%_^0%^, and is considered a standard. In this study, ventilation distributions using other phases were calculated and compared to this standard. To streamline comparisons, the base phase was always the end-expiration one (50%). A ventilation distribution calculated using an arbitrary phase *x* and the end-expiration phase can be further denoted as *V*_50%_^*x*^, where *x* = 10%, 20%, 30%, and so forth. A series of ventilation distribution data in a respiration cycle can also be considered a dynamic ventilation dataset.

Errors in DIR caused by the image artifacts and noise will be reflected in the calculated ventilation distributions [16]. To reduce their influence, the calculated ventilation datasets were smoothed with a 9 × 9 × 9 mm^3^ (or 9 × 9 × 3 voxels) average filter [10].

### 2.4. Correlation and Similarity

The voxel-wise nonparametric Spearman correlation coefficients (SCC) between the ventilation datasets, *V*_50%_^0%^ and *V*_50%_^*x*^, *x* = 10%, 20%, 30%,…, and also between neighboring phases, *V*_50%_^*x*−10%^ and *V*_50%_^*x*^, were calculated.

Dice similarity coefficient (DSC) [21], defined as

(2) $$\begin{array}{c} DSC\left(\;A,B\;\right)=\frac{2\times \left|A\cap B\right|}{\left|A\right|+\left|B\right|}, \end{array}$$

where *A* and *B* are the two involved volumes, was applied for the upper/lower 10%, 20%, 30% 40%, and 50% ventilation regions between pairs of ventilation datasets, including the original and smoothed data.

## 3. Results

**Figure 2:**
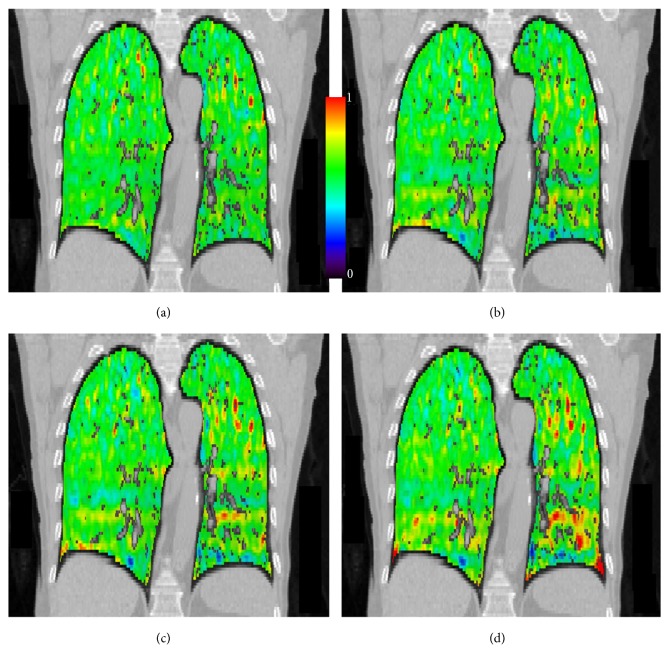
Coronal view of ventilation distributions for a typical case: (a) *V*_50%_^40%^, (b) *V*_50%_^30%^, (c) *V*_50%_^20%^, and (d) *V*_50%_^0%^ full ventilation. The color bar scale in the figure applies to all panels.

Figure 2 demonstrates a typical case of ventilation distribution calculated using deformable image registration and 4DCT. Four distributions are shown: *V*_50%_^40%^, which is calculated using the phase closest to end-expiration (50%) phase, *V*_50%_^30%^, *V*_50%_^20%^, and *V*_50%_^0%^, which corresponds to the full ventilation distribution.

**Figure 3:**
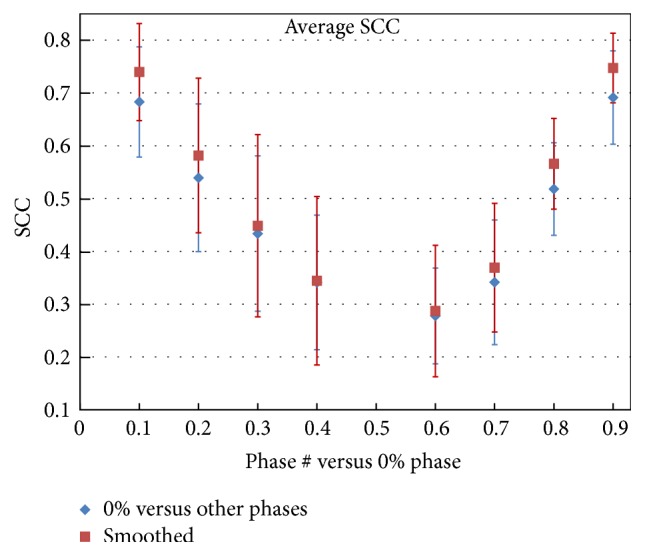
Average SCC value versus ventilation phase. Each SCC value represents the correlation between the full ventilation (*V*_50%_^0%^) and another phase *V*_50%_^*x*^ (ventilation calculated based on the *x* phase and 50% phase). The horizontal values represent *x* in *V*_50%_^*x*^.

Figure 3 shows the average SCC values for the ventilation distributions comparison between the full respiration *V*_50%_^0%^ and other phases *V*_50%_^*x*^, *x* = 10%, 20%, 30%,… in the dynamic ventilation datasets. All SCC comparisons were statistically similar (*p* < 0.0001). Note how the similarity was high when the second phase was close to the end-inspiration phase (*V*_50%_^10%^, *V*_50%_^90%^) and decreased for the phases closer to end-expiration (base) phase. The average SCC value for the original datasets was 0.68 ± 0.10 between *V*_50%_^0%^ and *V*_50%_^10%^, while it was 0.74 ± 0.09 after smoothing. For all the phases, the average SCC values were between 0.28 and 0.70 for the original datasets and between 0.30 and 0.75 after smoothing.

**Figure 4:**
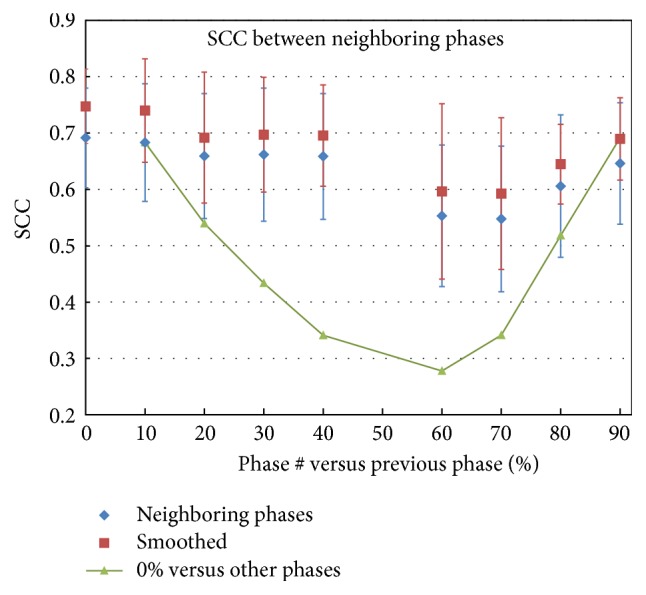
Average SCC values for neighboring phases. Each SCC value in this figure represents the correlation between ventilation distributions calculated based on the *x* phase *V*_50%_^*x*^ and the previous phase *V*_50%_^*x*−10%^. For example, SCC for 30% means the correlation between *V*_50%_^30%^ and *V*_50%_^20%^.

Figure 4 shows the average SCC values for the neighboring phases in the dynamic ventilation datasets. Each data point represents the SCC between the corresponding phase *V*_50%_^*x*^ and the previous phase *V*_50%_^*x*−10%^. For example, data point at 20% is the SCC between phases *V*_50%_^20%^ and *V*_50%_^10%^. There are two special data points that need further explanation. The point at 0% is the SCC between *V*_50%_^0%^ and *V*_50%_^90%^; the point at 60% is the correlation between *V*_50%_^60%^ and *V*_50%_^40%^. There is no data point at 50% because it is the base phase. Compared to the plot of the SCC values between full ventilation *V*_50%_^0%^ and other phases *V*_50%_^*x*^,  *x* = 10%, 20%, 30%,…  (Figure 3, redrawn in Figure 4 for comparison), the SCC values for the neighboring phases were flat. The average SCC values ranged between 0.55 and 0.70 for the original datasets and between 0.60 and 0.75 after smoothing.

**Figure 5:**
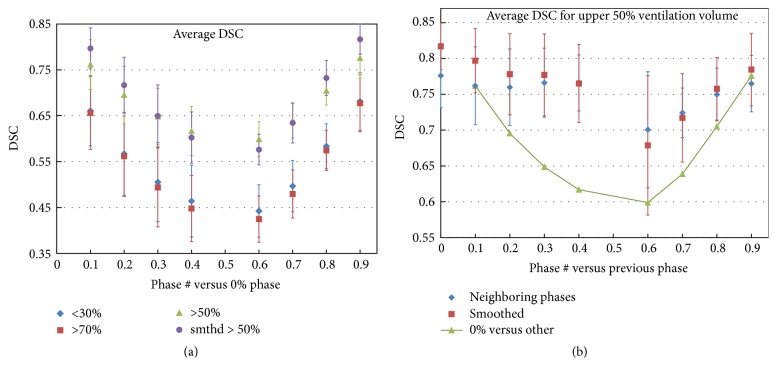
Dice similarity coefficient (DSC) data: (a) the average DSC versus phase for the lower 30%, upper 70%, and 50% ventilation volumes and (b) the average DSC for the 50% ventilation volume between neighboring phases. The solid line curve in (b) is redrawing of the DSC versus phase for the 50% volume as a reference.

Figure 5 shows the DSC results. Similar to the SCC results, the similarity of ventilation calculated with various phases versus the full ventilation was getting worse as the phase moved farther away from full inspiration, while the similarity between the neighboring phases was nearly flat.

The DSC values of other ventilation regions, including <10%, <20%, >80%, >90%, and so forth, were also calculated (data not presented). The DSC versus phase trends for all the ventilation regions were similar to that shown in Figure 5.

**Figure 6:**
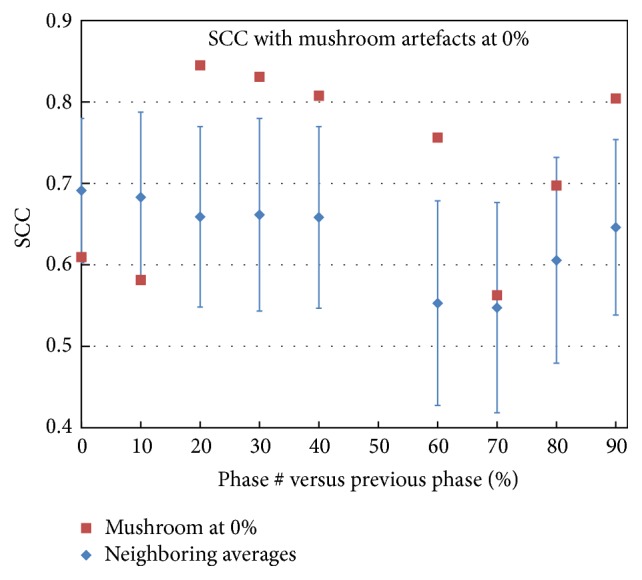
SCC comparison between a case with mushroom artifacts and the average values. All SCC values for the artifacts case with *V*_50%_^0%^ involved were lower than the corresponding average values. Note: 0% and 10% data in the figure correspond to SCC between *V*_50%_^90%^–*V*_50%_^0%^ and *V*_50%_^0%^–*V*_50%_^10%^.

For the case with mushroom artifacts in the end-expiration (0%) phase shown in Figure 1, the SCC values of *V*_50%_^0%^–*V*_50%_^10%^ and *V*_50%_^0%^–*V*_50%_^90%^ were 0.58 and 0.61, respectively, lower than the average values 0.68 and 0.69 (Figure 6). On the other hand, the SCC value of *V*_50%_^10%^–*V*_50%_^20%^ was 0.85, substantially higher than the corresponding average SCC value for all cases (0.66), indicating that the mushroom artifacts in the 0% phase introduced significant registration errors which caused low SCC of *V*_50%_^0%^–*V*_50%_^*x*^,  *x* = 10%, 20%, 30%,…. Using phases without mushroom artifacts can improve accuracy, resulting in higher similarity between calculations using different phases. Smoothed data (not presented) showed the same trend.

## 4. Discussion

The correlation and similarity comparison used in this study are only useful for relative ventilation distribution comparisons and not for absolute ventilation (or magnitude of the ventilation) differences. The magnitude of ventilation is certainly different between *V*_50%_^0%^ and *V*_50%_^*x*^, where *x* ≠ 0%. SCC compares the voxel-wise order of ventilation magnitude and DSC compares relative ventilation volumes between ventilation datasets, both with nothing to do with the absolute magnitude difference between the datasets. Clinically, the parameter of interest is the relative ventilation distribution; that is why different imaging modalities can be applied for the same purpose although what is measured in different imaging modality is different.

The poor similarity between the standard calculation (*V*_50%_^0%^) and those based on the ones close to end-expiration phase is likely due to the ventilation distribution being nonlinear with respect to phase, or the ventilation change rate for the same location being not consistent between the phases. As a result, ventilation distributions differ somewhat depending on the respiration phase used. This is supported by the data shown in Figures 4 and 5. The ventilation distributions based on neighboring phases were closely correlated to each other while the accumulated ventilation gradually diverged from the initial one, *V*_50%_^0%^, consistent with ventilation distribution slowly shifting with phase. Both the overall SCC values and subvolumes' DSC values showed the same trends. The subvolumes' DSC analysis demonstrated that all ventilation levels, from 10% through 90% (only partial results were presented in Results), had the similar nonlinearity property.

Based on Figures 4 and 5, the ventilation correlation/similarity between the 60% and 40% phases was the lowest, with the largest standard deviation. The reason for this is that patients tend to hold their breath at end-expiration for a short time, which makes the 60% phase very close to 50% (end-expiration phase), and the small difference between the two phases may cause relatively large image registration errors which in turn affect the ventilation distribution. The other reasons for reduced similarity may be noise and motion artifacts in the 4DCT data, which can have more pronounced effect on the registration between the neighboring phases. As a consequence, the correlation between *V*_50%_^60%^ and *V*_50%_^40%^ was the lowest with the largest standard deviation. To avoid the effect of motion artifacts in ventilation calculations, if there were artifacts present in the phases close to end-inspiration, one should use the 30% or 40% instead of the 60% or 70% phases. Slightly better correlation of *V*_50%_^40%^–*V*_50%_^0%^ than *V*_50%_^60%^–*V*_50%_^0%^ and of *V*_50%_^30%^–*V*_50%_^0%^ than *V*_50%_^70%^–*V*_50%_^0%^ can be also observed in Figure 3.

Smoothing of the ventilation distributions makes the correlation better in general, although occasionally, especially for the low correlation phases, smoothing may make the correlation worse. The reason for this phenomenon is most likely that smoothing can reduce the errors induced by image noise but not by other factors, for example, nonlinear change in ventilation. This can explain why, in Figures 3 and 5, the smoothing does not show any improvement in correlation between *V*_50%_^0%^–*V*_50%_^40%^ and *V*_50%_^60%^–*V*_50%_^70%^.

The results presented in Figure 6 demonstrate that the mushroom artifacts certainly affect the ventilation calculation, and using the artifact-free phases indeed improves the ventilation accuracy, as demonstrated by the enhanced similarity between different phases. Thus using the phases without mushroom artifacts, especially when close to end-inspiration, is conducive to getting more accurate ventilation distributions. However, the limitation of this study was that no clinically accepted ground truth was applied in the comparisons. The accuracy was determined only based on the series similarity of the 4DCT derived ventilation data. Further investigation is still needed to confirm the accuracy that resulted in this study.

This study attempts to improve ventilation calculations when obvious motion artifacts are present in 4DCT images. However the best approach would be to reduce or eliminate those artifacts in the first place. Amplitude-based binning, as opposed to the traditional phase-based binning, could reduce, but not completely eliminate, motion artifacts in 4DCT [22, 23]. Therefore the approach described in this study is useful regardless of the binning technique.

## 5. Conclusions

Motion artifacts are inevitably present in realistic 4DCT datasets. To reduce errors in the ventilation distributions caused by these artifacts, the respiratory phases that do not show serious mushroom artifacts should be used, as the correlation between the ventilation distributions obtained with different phases is reasonably good. To minimize the effect of the nonlinearity in dynamic ventilation, when the mushroom artifacts preclude the use of the end-inspiration phase, the phase used in the calculation should be as close to the end-inspiration as possible.
